# Developing a laboratory-based score to predict mortality in patients admitted to the ICU

**DOI:** 10.1186/cc14627

**Published:** 2015-03-16

**Authors:** A Iqbal, I Welters, R Kolamunnage-Dona, C Toh, C Downey

**Affiliations:** 1 Institute of Ageing and Chronic Disease, Liverpool, UK; 2 Royal Liverpool University Hospital, Liverpool, UK; 3 University of Liverpool, UK

## Introduction

Scoring systems can be used to predict mortality in patients admitted to the ICU. They are produced using variables that are associated with an increased risk of mortality such as patient demographics, physiological measurements and coexisting conditions and can be used to evaluate ICU performance, to stratify patients in clinical trials and to assist in-hospital and healthcare decisions such as resource allocation. The aim of the project was to determine whether a general score derived from routine laboratory parameters could be used to predict mortality rates in patients admitted to the ICU in the UK.

## Methods

*P *values were calculated using the *t *test, Mann-Whitney U test and chi-squared test, depending on distribution of data, in order to determine which variables were significantly different in the survivors and nonsurvivors of critical illness. Significant variables were categorised into subgroups according to medically relevant landmarks and univariately analysed by assessing the correlation with mortality. Forward logistic regression models were used to choose the parameters to include in our score. ROC curves illustrated the sensitivity and specificity of selected variables via their AUC.

## Results

Age, platelets, ALT and APACHE II were selected to be included in the new laboratory-based score. The AUC for the score was 0.714, which was higher than each of the individual laboratory parameters. The AUC was increased further to 0.781 by including all 14 variables (age, lactate, FiO_2_, urea, creatinine, ALT, APACHE II, platelet, bicarbonate, haemoglobin, pH, ionised Ca, carboxyhaemoglobin and albumin), although this improvement was not considered significant as the confidence intervals of the two scores (4 and 14 variables) overlapped.

## Conclusion

A laboratory-based score was successfully established in ICU patients, revealing an AUC of 0.714 which is comparable with established scores in a similar population. The compilation of the variables to produce a laboratory-based score showed greater prognostic power than individual variables. Model developers require an AUC of >0.7 to be termed useful; however, in order to be used in a clinical setting the AUC must be at least 0.75. Further research including internal and external validation studies must be performed to optimise the model before clinical implementation.

